# Dietary Sources of Lutein and Zeaxanthin Carotenoids and Their Role in Eye Health

**DOI:** 10.3390/nu5041169

**Published:** 2013-04-09

**Authors:** El-Sayed M. Abdel-Aal, Humayoun Akhtar, Khalid Zaheer, Rashida Ali

**Affiliations:** 1 Guelph Food Research Centre, Agriculture and Agri-Food Canada, Guelph, ON N1G 5C9, Canada; E-Mail: humayoun.akhtar@agr.gc.ca; 2 Consultant, Toronto, ON M3M 2E9, Canada; E-Mail: kzaheer2000@gmail.com; 3 Department of Food Science and Technology, ICCBS, University of Karachi, Karachi 75270, Pakistan; E-Mail: rashida338@gmail.com; 4 English Biscuit Manufacturers Pvt. Ltd., Korangi Industrial Area, Karachi 74900, Pakistan

**Keywords:** lutein and zeaxanthin carotenoids, dietary sources, eye health, high-lutein foods, bioavailability

## Abstract

The eye is a major sensory organ that requires special care for a healthy and productive lifestyle. Numerous studies have identified lutein and zeaxanthin to be essential components for eye health. Lutein and zeaxanthin are carotenoid pigments that impart yellow or orange color to various common foods such as cantaloupe, pasta, corn, carrots, orange/yellow peppers, fish, salmon and eggs. Their role in human health, in particular the health of the eye, is well established from epidemiological, clinical and interventional studies. They constitute the main pigments found in the yellow spot of the human retina which protect the macula from damage by blue light, improve visual acuity and scavenge harmful reactive oxygen species. They have also been linked with reduced risk of age-related macular degeneration (AMD) and cataracts. Research over the past decade has focused on the development of carotenoid-rich foods to boost their intake especially in the elderly population. The aim of this article is to review recent scientific evidences supporting the benefits of lutein and zexanthin in preventing the onset of two major age-related eye diseases with diets rich in these carotenoids. The review also lists major dietary sources of lutein and zeaxanthin and refers to newly developed foods, daily intake, bioavailability and physiological effects in relation to eye health. Examples of the newly developed high-lutein functional foods are also underlined.

## 1. Introduction

Nutrition plays a vital role in human health with no exception to the eye. Healthy eyes provide good vision, which is essential for an enjoyable and productive lifestyle. There is a growing global concern about eye health related issues. To address these issues the World Health Organization (WHO) report on visual impairment in 2010 identifies the principal causes of visual impairment as follows: uncorrected refractive errors (43%), cataracts (33%), glaucoma (2%), age-related macular degeneration (AMD) (1%), diabetic retinopathy (1%) and about 18% are of undetermined nature [1]. The report also lists the three major causes of blindness as cataract (51%), glaucoma (8%), AMD (5%), diabetic retinopathy (1%) and undetermined causes (21%). Cataract is the principal cause of blindness among people over 40 years of age predominantly in developing countries due to improper nutrition (e.g., lack of carotenoids in diet), infectious diseases [2]. AMD, on the other hand, is the leading cause of legal blindness with limited treatment options in people over 65 years of age in industrial countries, and costs many billions of dollars worldwide [2]. There are two types of AMD—dry (atrophic) and wet (neovascular or exudative). In most cases AMD starts as “Dry”, which then progresses slowly in approximately 20% cases to “Wet” stage. There is no known treatment for dry AMD. The wet AMD is responsible for almost 90% of the severe cases of blindness [3]. The prevalence of AMD is estimated to be a one-third increase in UK [4] and a 50% increase in USA by 2020 [5]. A similar estimate is also reported for Australia [6]. Both diseases are expected to sharply increase in the elderly population within the next 10–15 years. The Vision 2020, a global initiative for the elimination of avoidable blindness in partnership between WHO and International Agency for the Prevention of Blindness [IAPB] projects blindness due to cataracts alone in elderly to reach 40 million globally by 2025 [7]. It is clear that AMD and cataract incidences will continue to rise substantially in the coming years causing a great impact on health care. This requires global collective efforts to develop strategies for the prevention of the two most important common age-related diseases with appropriate diets/supplements. Several high-lutein functional foods that would be useful in developing such preventive strategies are discussed in the current article.

Oxidative stress, aging and smoking are known to cause cataract and AMD [8,9]. A typical US diet contains 1–3 mg/day of lutein and zeaxanthin, while ~6 mg/day have been related to decrease risk of AMD [8]. Lutein and zeaxanthin have been associated with reduced risk of cataract development and AMD [10]. A longitudinal study has shown that plasma zeaxanthin reduces the risk of cataract [11]. Hence lutein and zeaxanthin, both potent antioxidants, are very important to retard the onset of both cataract and AMD [12]. Additional sources of lutein and zeaxanthin either in the food form or dietary supplements would enhance their intake on regular basis. There are also reports that do not support the protective role of dietary lutein and zeaxanthin against cataracts [13] and AMD [14]. Recently an article that traces the modern history of lutein and zeaxanthin in health and diseases of retina identified four areas for further investigation: (i) ultra-structural localization of xanthophyll affected proteins in the retina, (ii) genetic analysis-genotyping efforts, (iii) model system for the metabolism of lutein and zeaxanthin and (iv) integrated system based approaches to trace the fate of lutein and zeaxanthin, its precursor(s) and metabolites [15].

This review article mainly focuses on lutein and zeaxanthin carotenoids in terms of their sources of common and newly developed foods, daily intake, bioavailability and physiological effects in relation to eye health. Examples of the newly developed high-lutein functional foods are underlined as well.

## 2. Lutein and Zeaxanthin Carotenoids

### 2.1. Chemistry

**Figure 1:**
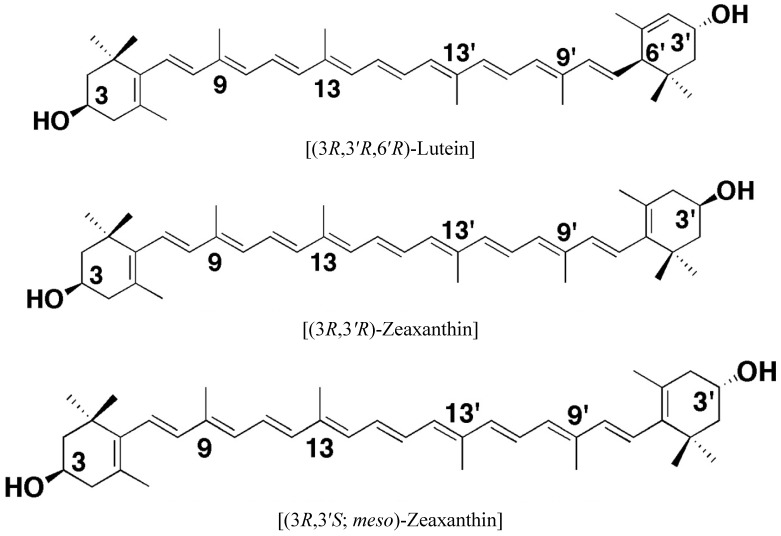
Chemical structures of macula pigments in retina.

Lutein and zeaxanthin are relatively polar carotenoid pigments found at high levels in parsley, spinach, kale, egg yolk and lutein-fortified foods. They have demonstrated several beneficial health effects due to their ability to act as scavengers for reactive oxygen species and to bind with physiological proteins in humans [16]. In general, carotenoids are tetra-terpenoid having 40 carbon skeleton made up of 8 isoprene units and comprise of two classes, namely carotenes (purely unsaturated hydrocarbons) and carotenoids with oxygen atoms which are referred to as oxygenated carotenoids or xanthophyll carotenoids. The macular carotenoids are dietary lutein and zeaxanthin, and their conversion isomer *meso*-zeaxanthin, which are non-provitamin A carotenoids, (*i.e.*, it cannot be converted into vitamin A). Important members of oxygenated carotenoids are lutein, zeaxanthin, β-cryptoxanthin, capsanthin, astaxanthin, and fucoxanthin. Figure 1 lists the chemical structure of the macular pigments found in the retina. It is estimated that about 90% of the total carotenoids in North American diets consist of lycopene, β-carotene, α-carotene, lutein, β-cryptoxanthin and zeaxanthin [17]. In nature more than 600 carotenoids have been isolated and characterized, yet only about 40 carotenoids have been detected in human milk, serum and tissues. Percentage of main carotenoids inhuman serum is lutein (20%), lycopene (20%), β-carotene (10%); β-cryptoxanthin (8%), α-carotene (6%) and zeaxanthin (3%) [18,19]. Lutein and zeaxanthin are the main dietary carotenoids found in human retina [20] and they protect the macula from damage by blue light, improve visual acuity and scavenge harmful reactive oxygen. Lutein and zeaxanthin along with their common metabolite *meso*-zeaxanthin, commonly referred to as macular pigments (MP) [21]. The ratio between lutein, zeaxanthin and *meso-*zeaxanthin changes as the eccentricity moves away from fovea [21,22,23]. Although lutein and zeaxanthin were also detected in prenatal eyes, they did not form visible yellow spot. No age-related (between the ages of 3 and 95 years) differences were observed in the quantity of lutein and zeaxanthin [21]. But, the ratio of lutein to zeaxanthin differed between infants and adults. In infants, lutein predominates over zeaxanthin in fovea, and the opposite is true after 3 years of age [21,24]. Structurally the difference between lutein and zeaxanthin is in the type of ionone ring, lutein contains a β-ionone ring and a ε-ionone ring, whereas zeaxanthin has two β-ionone rings. Lutein and zeaxanthin are isomers, but not stereoisomers, which differ in the location of a double bond unsaturation in the end ring (Figure 1). Lutein can exist in possible eight stereoisomeric forms because of three chiral centers, but in nature it exists mainly in Z (*cis*)-form (*R*,*R*,*R*). Zeaxanthin, on the other hand, has two chiral centers but, because of symmetry exists only in 3-stereoisomeric forms (*R*,*R*), (*S*,*S*) and (*R*,*S*-*meso*) (Figure 1).

### 2.2. Dietary Sources

Lutein and zeaxanthin are the most common xanthophylls in green leafy vegetables (e.g., kale, spinach, broccoli, peas and lettuce) and egg yolks [25] (Table 1). They are also found at relatively high levels in einkorn, Khorasan and durum wheat and corn and their food products [26,27,28,29] (Table 1). The ratio of lutein and zeaxanthin in green vegetables has been reported to range between 12 to 63, highest being in kale, while in yellow-orange fruits and vegetable this ratio ranges between 0.1 and 1.4 [30]. They also quantified small amounts of lutein and zeaxanthin in breads prepared from modern wheat varieties, Pioneer and Catoctin, while breads prepared from green-harvested wheat, Freekeh, an ancient grain, contained considerably large amounts of lutein and zeaxanthin compared to the North American breads. Lutein to zeaxanthin ratio followed the order Pioneer > Catochtin > Freekeh [30]. Chicken egg yolk is deemed a better source of lutein and zeaxanthin compared to fruits and vegetables because of its increased bioavailability due to the high fat content in eggs [31,32]. The concentrations of lutein and zeaxanthin in chicken egg yolk are 292 ± 117 µg/yolk and 213 ± 85 µg/yolk (average weight of yolk is about 17–19 g), respectively and are likely dependent on the type of feed, found mainly in on-esterified form with minute amounts of lycopene and β-carotene [33]. It is not surprising that egg noodle had almost 6 times more xanthophyll carotenoids than lasagne [30]. Astaxanthin and fucoxanthin are abundant in green and brown algae, respectively, which are eaten by fish. Capsanthin is found mainly in pepper. β-Cryptoxanthin is a pro-vitamin A and found in many fruits and vegetables, but mainly in corn, oranges, peaches, papaya, watermelon, and egg yolk [34,35].

**Table 1:** Selected commonly consumed foods as high sources of xanthophylls (µg/g fresh weight except for corn tortilla and chips µg/g dry matter) [25].

Food	Lutein	Zeaxanthin
Vegetables		
Basil ^a^	70.5	in
Parsley ^a^	64.0–106.5	in
Spinach ^a^	59.3–79.0	in
Kale ^a^	48.0–114.7	-
Leek ^a^	36.8	in
Pea ^a^	19.1	in
Lettuce ^a^	10.0–47.8	-
Green pepper ^a^	8.8	-
Broccoli ^a^	7.1–33.0	in
Carrot ^a^	2.5–5.1	in
Red pepper ^a^	2.5–85.1	5.9–13.5
Eggs		
Egg yolk ^a^	3.84–13.2	-
Nuts		
Pistachio ^a^	7.7–49.0	-
Baked foods		
High lutein bread ^b^	36.7	3.3
High lutein cookie ^b^	21.3	2.9
High lutein muffin ^b^	26.1	3.7
Corn tortilla ^c^	72.5	105.3
Corn chips ^c^	61.1	92.5
Grains		
Corn ^d^	21.9	10.3
Einkorn wheat ^d^	7.4	0.9
Khorasan wheat ^d^	5.5	0.7
Durum wheat ^d^	5.4	0.5

Data obtained from: a, [26]; b, [27]; c, [28]; d, [29]; in = included with lutein.

In general carotenoids are very minor constituents in cereal grains except for einkorn and durum wheat and corn that contain relatively high levels of carotenoids or yellow pigments [27,29,36]. The common carotenoids in cereal grains are α and β-carotene, β-cryptoxanthin, lutein and zeaxanthin with lutein being the dominant carotenoid compound. In common wheat flour (low in carotenoids), the bran/gem fraction had 4-fold more lutein, 12-fold more zeaxanthin, and 2-fold more β-cryptoxanthin than the endosperm fractions [37]. Higher amounts of lutein were found in durum, Kamut and Khorasan (5.4–5.8 µg/g) compared with common bread and pastry wheat (2.0–2.1 µg/g). Einkorn, on the other hand, had the highest concentration of all-*trans*-lutein, which is influenced by environmental growing conditions [29] and processing [27]. Corn also contains exceptionally high levels of non-provitamin A carotenoids primarily lutein and zeaxanthin [29,38].

### 2.3. Bioavailability

In order to exert and deliver their physiological effects carotenoids must be absorbed and transported into the blood stream. In general, carotenoids are lipophilic or hydrophobic which are soluble in fat and insoluble in aqueous media, the medium of human digestive system. Because of the hydroxyl groups, lutein and zeaxanthin are polar compounds compared with the hydrocarbon carotenoids (α-, β-carotene, and lycopene). Thus, a good understanding of carotenoid release, absorption, transportation and accumulation in eye is essential to evaluate the benefits. 

The major factors that influence the absorption of carotenoids including lutein and zeaxanthin from food include (i) nature of the food matrix, e.g., in natural format, cooked or supplement, (ii) amount and nature of the dietary fat, which aids in the solubilisation of released carotenoids, (iii) phospholipids, (iv) dietary fiber, (v) nature of carotenoids [39,40,41]. The absorption of carotenoid released from food include several steps (i) dispersion in the gastric emulsion to be incorporated into lipid droplets, (ii) followed by transfer to mixed micelles involving bile salts, biliary phospholipids, dietary lipids and others. Solubilized carotenoids are then absorbed by the intestinal cell for transportation into blood system. These steps may include simple diffusion, uptake by micelles and receptor mediated and other transporter, as schematically presented by Nagao and colleagues [42,43]. The highest concentration of carotenoids in micelles (*i.e.*, solubilisation), corresponds to greater absorption and transportation into plasma. In general, bioavailability of carotenoids is affected by a number of factors including food matrix, processing conditions and fat content [39,44], while the rate of bio-accessibility of carotenoids is greatly impacted by food matrix and processing. It was observed that the in vitro rate of lutein, zeaxanthin and β-cryptoxanthin transfer almost 100% from fruits (orange, kiwi, grapefruit and sweet potato) compared to between 19% and 38% from spinach and broccoli, respectively [45]. The release of carotenoids from a food matrix followed by absorption is the determining factors for delivering the anticipated health benefits. Since carotenoids are found in a food matrix, they could be released prior to consumption by processing and heat treatment [46]. In other words, the intestinal absorption and metabolic transformation determine the efficacies of carotenoids including transportation and accumulation of macula pigments (MP) in retina that leads to protection of retina, possible prevention and/or slowing the progress of blindness.

Dietary lutein and zeaxanthin and the metabolite *meso*-zeaxanthin are concentrated (~25% of the total carotenoids) in the macula region of healthy eye as a yellow spot, but considerably less in deceased eyes [47,48,49]. *meso*-Zeaxanthin a non-dietary carotenoid is not found in serum, but only in retina. It has been suggested that lutein and zeaxanthin are transported into retina in the same ratio as in plasma, and then transferred to macula where lutein is preferentially converted into *meso*-zeaxanthin [47,48,49,50,51]. These observations strongly suggest the importance of lutein, zeaxanthin and *meso*-zeaxanthin in the management of good eye health [51,52,53]. Several studies have shown that incidences of AMD can be reduced by consuming diets with high levels of lutein and zeaxanthin and supplementation by increasing their concentration in serum and parallel increase in macular pigment optical density (MPOD) [54,55,56,57,58]. For example, lutein supplementation over a 140-day period increased serum lutein level [55]. Similarly, consuming increased spinach and kale in diet for a 4-week period increased the MPOD by 4%–5%. Recently, a systematic review and meta-analysis of several longitudinal studies have concluded that lutein and zeaxanthin affect positively in the case of late AMD but not early AMD [59]. The early or dry AMD was defined by the presence of drusen pigment abnormalities in retina pigment epithelium (RPE) or both whereas the late or wet AMD includes neovascular AMD and geographic atrophy by the presence of choroidal neovascularization, detachment of RPE or geographic atrophy [59].

Concentration and nature of various carotenoids in eye (macula and retina) were established in early 1990. In the fovea, the carotenoid concentration approaches 1 mM, and the ratio of lutein to zeaxanthin to *meso*-zeaxanthin is 1:1:1 [47]. The concentration of macular carotenoids declines over 100-fold just a few millimeters from the foveal center, and the composition ratio approaches 3:1:0 in the peripheral retina (around 21 mm) *i.e.*, lutein concentration is considerably higher with no zeaxanthin in peripheral retina [47,50,60]. The exclusiveness (>80% of total carotenoids) of lutein and zeaxanthin in retina from among 40 carotenoids found in serum is unique and intriguing since the presence of about 20%–25% of total carotenoids in human plasma cannot account for by routine transportation systems [61,62]. In general, carotenoids are transported mainly by low-density lipoprotein (LDL, 55%), followed by high-density lipoprotein (HDL, 33%) and very low density lipoprotein (VLDL, 10% to 19%) and others [63]. However, lutein and zeaxanthin are distributed equally in LDL and HDL, with more infinity towards HDL [64]. These data suggest that lipoprotein profile of serum would play important role in transport and concentration of lutein and zeaxanthin in retina and MPOD. A study reported negative relationship between serum triglycerides and MP, and no relations between MPOD and HDL and LDL [65]. The authors also observed lower than the normal serum lutein and zeaxanthin levels. On the other hand, another study found positive relationships between serum lutein and zeaxanthin with total cholesterol and HDL, inversely related to triacylglycerols, but none with LDL [58]. A most recent cross-sectional study observed that serum lutein and zeaxanthin and lipoprotein concentrations are significantly related [66]. The study concludes that changing lipoprotein concentrations may impact retinal lutein and zeaxanthin levels. Evidences for HDL primary role in the transport of lutein and zeaxanthin were provided when it was reported that feeding high lutein diet to the Wisconsin Hypoalpha Mutant Chicken deficient in HDL increased lutein concentration significantly in various tissues/organ except in retina [67]. Egg, a rich source of lutein and zeaxanthin, is an integral part of the American diet, but with continued concerns for increasing serum lipids and lipoproteins concentrations. A randomized cross-over design study involving 33 men and women consuming 1 egg per day for 5 weeks reported increased serum lutein (26%), and zeaxanthin (38%), but serum concentrations of total cholesterol, LDL cholesterol, HDL cholesterol and triacylglycerols were not affected [68]. Most recently it was reported that daily intake of 3 eggs for 12 weeks increased the lutein and zeaxanthin by 21% and 48%, respectively in 20 adults [69]. Thus, egg yolk could be an important dietary source to improve lutein and zeaxanthin status for the prevention of cataracts and AMD in adults. Similarly, a separate study with simultaneous administration of large amounts of lutein, zeaxanthin and β-carotene affected their concentrations in plasma, tissues and retina [70]. The retinal concentration of lutein and zeaxanthin increased 128% and 116% respectively, when fed diet with high lutein (27.2 mg/kg) and zeaxanthin (15.3 mg/kg) for 28 days compared to the control diet with lutein (5.2 mg/kg) and zeaxanthin (1.7 mg/kg). Further, it was observed that supplementation of β-carotene increased its value in plasma very little, and none in the other tissues and retina. However, high supplementation of β-carotene in conjunction with high lutein and zeaxanthin diet decreased lutein and zeaxanthin concentration in plasma, tissue and retina [70]. The mechanism is not well understood. A recent explanatory study using a formulation consisting of all the three MP carotenoids (7.3 mg *meso*-zeaxanthin, 3.7 mg lutein and 0.8 mg zeaxanthin) over an 8 week period significantly increased the serum concentration of these carotenoids as well as the MPOD [71].

It is more likely that transportation and accumulation of lutein and zeaxanthin involve preferential and efficacious complex-binding with other circulating proteins. Such xanthophyll-lipoprotein complex(s) are then circulated in blood stream. It is interesting to note that blue colouration in lobster shell has been shown to be due to the complex-binding of carotenoid (astaxanthin) with a protein referred to as crustacyanin [72]. It would, therefore, be reasonable to explore similar complex-binding protein(s) in humans and other vertebrates that may be responsible for transportation of lutein and zeaxanthin to the macula. Bhosale and co-workers [73] reported that Pi isoform of glutathione transferase protein (GSTPi) in the human macula has a greater interaction with zeaxanthin and *meso*-zeaxanthin compared with lutein which has a weaker interaction. Subsequently, these researchers also purified and identified a lutein-binding protein in the macula of human eye labeled as StARD3 (Steroidogmic regulatory domain), also known as MNL64 [74,75]. Another team investigated the mechanism for preferential uptake of xanthophylls by retina pigment epithelial (RPE) cells. They reported that RPE cells preferentially take up xanthophylls than other carotenes by SR-BI dependent-dependent process(s) to preferentially accumulate in the macula of retina [76]. Overall, absorption and transportation of carotenoids in foods are complex multi-step processes.

### 2.4. Eye Health

Considerable research has been undertaken on global level to generate new knowledge to understand the causes, and define steps/processes to slow the onset of cataract and AMD through diets. Evidences show that lutein and zeaxanthin are important dietary carotenoids in preventing and reducing cataracts and AMD. A multi-center eye disease case-control study involving five ophthalmology centers in the US showed that a higher dietary intake of carotenoids, specifically lutein and zeaxanthin is associated with reduced AMD risk [8]. Thus, our efforts will be limited to reviewing the existing literature on the role of dietary carotenoids in reducing the incidences of cataract and AMD. In general, the ageing processes cause biochemical, physiological and physical changes that are directly or indirectly responsible for the onset of many diseases including cataract and AMD. The pathogeneses of both cataract and AMD formation are not fully established. However, over the years research has identified major contributing factors for both diseases. Ageing (greater than 50 years of age) seems to be the major cause in both cases. In addition, other factors include exposure to ultraviolet light (up to around 380 nm) and blue light (400–500 nm high energy), oxidative stress due to access of oxygen radical species, environmental factors, and high polyunsaturated fatty acids responsible for AMD [77]. High incidences of cataract have been linked to poverty and poor nutrition, and strict vegetarian diets lacking in antioxidants [78,79].

High macular pigment density (MPD), macular pigment optical density (MPOD) and macular pigment (MP) have been associated with reduced AMD. There are studies that have linked ethnicity to the development of AMD because of differences in the MPD, MPOD and MP distribution in retina among various races. For example, Wolf-Schnurrbusch and co-workers [80] recorded significantly higher MPD in African subjects compared to White non-Hispanic subjects (0.59 ± 0.14 DU *versus* 0.36 ± 0.13 DU). This provides scientific explanations for previously observed higher incidences of AMD in white non-Hispanic compared to African populations in East Baltimore, USA. Further, a reverse trend was observed for cataracts, *i.e.*, its incidence is 4 times higher in Blacks than White non-Hispanic subjects [81]. These observations have been supported by several other studies that have linked lutein and zeaxanthin concentrations to the reduction of cataracts in North Indian population [82], in older American women [83] and in Australian population [84]. Recent studies also reported the prevalence of major eye diseases and showed that high plasma concentrations of lutein and zeaxanthin reduced the risk of age-related cataract in the elderly Finnish population by about 41% [85,86]. Studies involving older Europeans from Norway, Estonia, United Kingdom, France, Italy, Greece and Spain have reported high AMD prevalence (4% to 12%) depending on age [87]. However, studies from China, India and Korea show a prevalence rate of around 4% [88,89,90]. The Beijing study [88] involved 4439 subjects over 40 years of age residing in rural and urban areas and the Korean studies had 10,449 subjects over 40 years [90]. These studies suggest less prevalence of AMD in Asians than Caucasians. In addition, several epidemiological studies have found a close relationship between dietary carotenoids, more specifically the amount of lutein and zeaxanthin, and the incidences of AMD [91,92,93]. 

## 3. High-Lutein Functional Foods

As mentioned earlier a number of wheat species such as einkorn (ancient wheat) and durum (pasta wheat) and corn hold a potential for developing high-lutein staple foods. These cereals were identified as promising ingredients for the development of high-lutein functional foods based on their relatively higher levels of lutein compared with other wheat species such as spelt, soft and hard wheat [94,95]. Lutein content ranges from 5.4 to 7.4 µg/g in high-lutein wheat species and about 21.9 µg/g in corn. Lutein and zeaxanthin are the major carotenoids in corn milled fractions and account for about 70% of the total carotenoids [96]. This makes corn a promising blending flour ingredient in the development of high-lutein functional foods.

Three wholegrain functional foods with high level of lutein (about 1 mg per 30 g serving) were developed and evaluated in terms of lutein stability during baking process [27], lutein digestibility in vitro using fasted and fed model [44], phenolic antioxidants [97], and antioxidant properties [98]. The wholegrain bakery products include high-lutein flat bread, high-lutein cookie and high-lutein muffin. Lutein was found to drop significantly during baking process (28% to 64% loss) due to oxidation and isomerization. A number of *cis*-isomers were found in the three products with 13- and 13′-*cis*-lutein being the dominant *cis*-isomers. Due to the significant losses of lutein a fortification approach was used to boost lutein in the functional food products and to compensate for the losses of lutein occurred during processing and/or storage. Other approaches could also be used such as protection of lutein during processing or developing wheat and corn verities with higher lutein content than the existing ones. Despite the significant losses of lutein during processing, the developed fortified baked products still contain reasonable concentrations (up to 1 mg/serving) of lutein and would hold a promise as high-lutein staple functional foods. Bioavailability of lutein in the wholegrain bread, cookie and muffin was also investigated using fasted and fed digestion model in which food products were subjected to an *in vitro* simulation of human salivary, gastric and duodenal digestion, and then followed by Caco-2 monolayer absorption [44]. The fed model resulted in much higher estimates of bioavailability of lutein and the higher fat products (cookie and muffin) resulted in higher overall bioavailability. Antioxidant capacity of the products varied among the baked products subject to product type, type of bioactive components (e.g., unbound *versus* bound phenolic compounds) and antioxidant assay [98]. In the ORAC assay similar antioxidant capacities were obtained for unbound phenol extracts either from fortified or unfortified high-lutein products, while significant differences were observed for bound phenol extracts. Significant differences were also found between unbound and bound phenol extracts in their ability to scavenge ABTS radical cation. In the DPPH assay lutein-fortified products had scavenging capacities significantly higher than that of the unfortified ones. In general, the bound phenolic extracts contribute significantly higher than the unbound phenol extracts to the antioxidant capacity. Only the DPPH test showed the contribution of lutein to the antioxidant capacity. The baking process was found to increase free phenolic acids in the three products, while bound phenolic acids was decreased in bread and slightly changed in cookie and muffin products [97]. Though the effect of baking appeared to be dependent on type of baked product, type of phenolic, recipe and baking conditions, the wholegrain products should be considered good sources of phenolic antioxidants.

Corn products are also rich in carotenoids primarily lutein and zeaxanthin and would be potential candidates for making cereal-based functional foods if high carotenoids varieties are chosen for processing. Lutein, zeaxanthin and other carotenoids in processed corn including canned corn, corn meal, corn flour and corn flake were found to vary between the products and between brands of the same product, but variations between lots of the same brand was small [28]. Among five corn types including white, yellow, high-carotenoid, blue and red corns, lutein content was highest in yellow corn (406 µg/100 g) and lowest in blue and white corns (5.2 and 5.7 µg/100 g, respectively) [99]. Lime-cooking significantly decreased lutein content in yellow, red and high-carotenoid corns. Further processing into tortillas and tortilla chips did not significantly affect lutein content except for yellow corn chips. The contents of lutein and zeaxanthin in corn tortilla and chips made from high-carotenoid variety are presented in Table 1.

## 4. Conclusions

Increasing age is the dominating factor for the onset of cataracts and AMD because of physiological and biochemical changes due to old age. Global researchers have identified lack of lutein and zeaxanthin as dietary causes in cataract and AMD related blindness. To date large scale prevalence studies provide serious data that conclude ethnicity may be the other major factors. But, age, diet and ethnicity are not the only factors for cataract and AMD. Hence prevention programs should not be solely based on these factors. Other factors such as living (geographic location) and working environment, socio-economic standing and hitherto unidentified factors should also be investigated. More research looking into the development of high-xanthophyll functional foods is essential in order to develop dietary strategies for the management of cataract and AMD in particular for elderly people.

## References

[1] World Health Organization Global data on visual impairments, 2012. http://www.WHO.int/blindness/GLOBALDATAFINALforweb.pdf.

[2] Gottlieb J.L. (2002). Age-related macular degeneration. JAMA.

[3] Mogk L. The differences between wet and dry age-related macular degeneration, 2013. http://www.visionaware.org/section.aspx?FolderID=6&SectionID=134&DocumentID=5972.

[4] Owen C.G., Tarrar Z., Wormald R., Cook D.G., Fletcher A.E., Rudnicka A.R. (2012). The estimated prevalence and incidence of late stage age-related macular degradation in the UK. Br. J. Ophthalmol..

[5] Friedman D.S., O’Colmain B.J., Munoz B. (2004). Prevalence of age-related macular degeneration in the United States. Arch. Ophthalmol..

[6] Taylor H., Guymer R., Keefe J., Limited A.E.P. (2006). The Impact of Age-Related Macular Degeneration.

[7] IAPB (The International Agency for the Prevention of Blindness) Vision 2020—The Right to Sight. http://www.iapb.org.

[8] Seddon J.M., Ajani U.A., Sperduto R.D., Hiller R., Blair N., Burton T.C., Farber M.D., Gragoudas E.S., Haller J., Mille D.T. (1994). Dietary carotenoids, vitamin A,C and E, and advanced age-related macular degeneration. Eye Disease Case-Control Study Group. JAMA.

[9] Richer S., Stiles W., Statkute L., Pulido J., Frankowski J., Rudy D., Pei K., Tsipursky M., Nyland J. (2004). Double-masked, placebo-controlled, randomized trial of lutein and antioxidant supplementation in the intervention of atrophic age-related macular degeneration: The Veterans LAST study (Lutein Antioxidant Supplementation Trial). Optometry.

[10] Basu H.N., Del Vacchio A., Flider F., Orthoefer F.T. (2001). Nutritional and potential disease prevention properties of carotenoids. J. Am. Oil Chem. Soc..

[11] Delcourt C., Carriere I., Delage M., Barberger-Gateau P., Schalch W. (2006). Plasma lutein and zeaxanthin and other carotenoids as modifiable risk factors for age-related maculopathy and cataract: The POLA Study. Invest. Ophthalmol. Vis. Sci..

[12] Tan J.S.L., Wang J.J., Flood V., Rochtina E., Smith W., Mitchell P. (2008). Dietary antioxidants and the long-term incidences of age-related macular degeneration—The Blue Mountains Eye Study. Ophthalmology.

[13] Lyle B.J., Mares-Perlman J.A., Klein B.E., Klein R., Patta M., Bowen P.E., Greger J.L. (1999). Serum carotenoids and tocopherols and incidence of age-related nuclear cataract. Am. J. Clin. Nutr..

[14] Cho E., Hankinson S.E., Rosner B., Willet W.C., Colditz G.A. (2008). Prospective study of lutein/zeaxanthin intake and risk of age-related macular degeneration. Am. J. Clin. Nutr..

[15] SanGiovanni J.P., Neuringer M. (2012). The putative role of lutein and zeaxanthin as protective agents against age-related macular degeneration: promise of macular genetics for guiding mechanism and translational research in the field. Am. J. Clin. Nutr..

[16] Snodderly D.M. (1995). Evidence for protection against age-related macular degeneration by carotenoids and antioxidant vitamins. Am. J. Nutr..

[17] Rao A.V., Rao L.G. (2007). Carotenoids and human health. Pharmacol. Res..

[18] Khachik F., Beecher G.R., Goli M.B. (1992). Separation and identification of carotenoids and their oxidation products in the extrcats of human plasma. Anal. Chem..

[19] Khachik F., Spangler C.J., Smith J.C., Canfield L.M., Steck A., Pfander H. (1997). Identification, quantification, and relative concentration of carotenoids and their metabolites in human milk and serum. Anal. Chem..

[20] Krinsky N.I., Landrum J.I., Bone R.A. (2003). Biological mechanisms of the protective role of lutein and zeaxanthin in the eye. Ann. Rev. Nutr..

[21] Bone R.A., Landrum J.T., Fernandez L., Tarsis S.L. (1988). Analysis of macular pigment by HPLC: Retinal distribution and age study. Invest. Ophthalmol. Vis. Sci..

[22] Handelmam G.J., Dratz E.A., Reay C.C., van Kuijk J.G. (1988). Carotenoids in the human macula and whole retina. Invest. Ophthalmol. Vis. Sci..

[23] Landrum J.T., Bone R.A. (2001). Lutein, zeaxanthin, and the macular pigment. Arch. Biochem. Biophys..

[24] Moukarzel A.A., Bejjani R.A., Fares F.N. (2009). Xanthophylls and eye health in infants and adults. J. Med. Liban..

[25] Perry A., Rasmussen H., Johnson E.J. (2009). Xanthophyll (lutein, zeaxanthin) content of fruits, vegetables and corn and egg products. J. Food Comp. Anal..

[26] Maiani G., Periago Caston M.J., Catasta G., Toti E., Cambrodon I.G., Bysted A., Granado-Lorencio F., Olmedilla-Alonso B., Knuthsen P., Valoti M. (2009). Carotenoids: Actual knowledge on food sources, intakes, stability and bioavailability and their protective role in humans. Mol. Nutr. Food Res..

[27] Abdel-Aal E.-S.M., Young J.C., Akhtar H., Rabalski I. (2010). Stability of lutein in wholegrain bakery products naturally high in lutein or fortified with free lutein. J. Agric. Food Chem..

[28] De La Parra C., Saldivar S.O.S., Lui R.H. (2007). Effect of processing on the phytochemical profiles and antioxidant activity of corn for production of masa, tortillas and tortilla chips. J. Agric. Food Chem..

[29] Abdel-Aal E.-S.M., Young J.C., Rabalski I., Frégeau-Reid J., Hucl P. (2007). Identification and quantification of seed carotenoids in selected wheat species. J. Agric. Food Chem..

[30] Humphries J.M., Khachik F. (2003). Distribution of lutein, zeaxanthin, and related geometrical isomers in fruits, vegetable, wheat, and pasta products. J. Agric. Food Chem..

[31] Mangels A.R., Holden J.M., Beecher G.R., Forman M.R., Lanza E. (1993). Carotenoid contents of fruits and vegetables—an evaluation of analytical data. J. Am. Diet. Assoc..

[32] Schaeffer T.L., Tyczkowski J.R., Parkhurst C.R., Hamilton P.B. (1988). Carotenoid composition of serum and egg yolk of hens fed diets varying in carotenoid composition. Poultry Sci..

[33] Handleman G.H., Nightingale Z.D., Lichtenstein A.H., Schaefer E.J., Blumberg J.P. (1999). Lutein and zeaxnathin concentrations in plasm after dietary supplementation with egg yolk. Am. J. Clin. Nutr..

[34] Chandrika U.G., Jansz E.R., Wickranasinghe S.M.D.N., Warnasuriya N.D. (2003). Carotenoids in yellow and red-fleshed papaya (*Carcia papaya* L). J. Sci. Food Agric..

[35] United States Department of Agriculture USDA Nutritional database for standard reference release 22, 2009. Nutritional data laboratory home page.

[36] Abdel-Aal E.-S.M., Young J.C., Wood P.J., Rabalski I., Hucl P., Fregeau-Reid J. (2002). Einkorn: A potential candidate for developing high lutein wheat. Cereal Chem..

[37] Adom K.K., Sorrells M.E., Liu R.H. (2005). Phytochemicals and antioxidant activity of milled fractions of different wheat varieties. J. Agric. Food Chem..

[38] Moros E.E., Darnoko D., Cheryan M., Perkins E.G., Jerrell J. (2002). Analysis of xanthophylls in corn by HPLC. J. Agric. Food Chem..

[39] Van Het Hof K.H., Weststrate J.A., Hautvast J.G. (1999). Dietary factors that affect the bioavailability of carotenoids. Nutr. Res..

[40] Castenmiller J.J., West C.E. (1998). Bioavailability and bioconversion of carotenoids. Annu. Rev. Nutr..

[41] Bohn T. (2008). Bioavailability of non-provitamin A carotenoids. Curr. Nutr. Food Sci..

[42] Yonekura L., Nagao A. (2007). Intestinal absorption of dietary carotenoids. Mol. Nutr. Food Res..

[43] Nagao A. (2011). Absorption and metabolism of dietary carotenoids. BioFactors.

[44] Read A. (2011). Influence of digestion model, product type and enrichment level on *in vitro* bioavailability of lutein from high lutein functional bakery products. M.Sc. Thesis.

[45] O’Connell O.F., Ryan L., O’Brien N.B. (2007). Xanthophyll carotenoids are more bioaccessible from fruits than dark green vegetables. Nutr. Res..

[46] Thurnham D.I. (2007). Macular zeaxanthins and lutein- a review of dietary sources and bioavailability and some relationships with macular optical density and age-related disease. Nutr. Res. Rev..

[47] Bone R.A., Landrum J.T., Friedes L.M., Gomez C.M., Kilburn M.D., Menendez E., Vidal I., Wang W. (1997). Distribution of lutein and zeaxanthin stereoisomers in the human retina. Exp. Eye Res..

[48] Bone R.A., Landrum J.T., Dixon Z., Chen Y., Llerena C.M. (2000). Lutein and zeaxanthin in the eyes, serum and diet of human subjects. Exp. Eye Res..

[49] Whitehead A.J., Mares J.A., Danis R.P. (2006). Macular pigment: A review of current knowledge. Arch. Ophthalmol..

[50] Bone R.A., Landrum J.T., Hime G.W., Cains A., Zamor J. (1993). Stereochemistry of the human macular carotenoids. Invest. Ophthalmol. Vis. Sci..

[51] Mozaffarieh M., Sacu S., Wedrich A. (2003). The role of the carotenoids lutein and zeaxanthin, in protecting against age-related macular degeneration: A review based on controversial evidence. Nutr. J..

[52] Bone R.A., Landrum J.T., Beatty S., Nolan J. (2011). Targeting AMD with a critical carotenoid. Rev. Ophthalmol..

[53] Albert G., Hoeller U., Schierle J., Neuringer M., Johnson E., Schaich W. (2008). Metabolism of lutein and zeaxanthin in Rhesus monkey: Identification of (3*R*,6′*R*)-, and (3*R*,6′*S*)-3′-hydro-lutein as common metabolites and comparison of humans. Comp. Biochem. Physisol. B Biochem. Mol. Biol..

[54] Hammond B.R., Johnson E.J., Russell R.M., Krinsky N.I., Yeum K.J., Edwards R.B., Snodderly D., Russell R.M. (1997). Dietary modification of human macular pigment density. Invest. Ophthalmol. Vis. Sci..

[55] Berendschot T.T.M., Goldbohn R.A., Klopping W.A.A., van der Kraats J., van Norel J., van Norren D. (2000). Influence of lutein supplementation on macular pigment assessed with two objective techniques. Invest. Ophthalmol. Vis. Sci..

[56] Johnson E.J., Hammond B.R., Yeum K.-J., Qin J., Wang X.D., Castaneda C., Snodderly D., Russell R.M. (2000). Relation among serum and tissue concentrations of lutein and zeaxanthin and macular pigment density. Am. J. Clin. Nutr..

[57] Landrum J.T., Bone R.A., Joa H., Kilburn M.D., Moore L.L., Sprague K.E. (1997). A one-year study on the macular pigment—the effect of 140 days of a lutein supplementation. Exp. Eye Res..

[58] Loane E., Nolan J.M., Beatty S. (2010). The respective relationships between lipoprotein profile, macular pigment optical density, and serum concentrations of lutein and zeaxanthin. Invest. Ophthalmol. Vis. Sci..

[59] Ma L., Dou H.-L., Wu Y.-Q., Huang Y.-M., Huang Y.-B., Zou Z.-Y., Lin X.-M. (2012). Lutein and zeaxanthin intake and the risk of age-related macular degeneration: A systematic review and meta-analysis. Br. J. Nutr..

[60] Khachik F., de Moura F.F., Zhao D.Y., Aebischer C.P., Bernstein P.S. (2002). Transformations of selected carotenoids in plasma, liver, and ocular tissues of humans and in nonprimate animal models. Invest. Ophthalmol. Vis. Sci..

[61] Handelman G.J., Snodderly D.M., Adler A.J., Russett M.D., Dratz E.A. (1992). Measurement of carotenoids in human and monkey reina. Methods Enzymol..

[62] Handelman G.J., Shen B., Kinsky N.L. (1992). High resolution analysis of carotenoids in human plasma by high performance liquid chromatography. Methods Enzymol..

[63] Clevendice B.A., Bieri J.G. (1993). Association of carotenoids with human plasma lipoproteins. Methods Enzymol..

[64] Goulinet S., Chapman M.J. (1997). Plasma LDL and HDL subspecies are heterogeneous in particle content of tocopherols and oxygenated and hydrocarbon carotenoids. Relevance to oxidative resistance and atherogenesis. Arterioscler. Thromb. Vasc. Biol..

[65] Broekmans W.M.R., Berendschot T.T.J.M., Klopping-Ketelaars I.A.A., de Vries A.J., Goldbohm R.A., Tijburg L.B.M., Kardinaal A.F.M., van Poppel G. (2002). Macular pigment density in relation to serum and adipose tissue concentrations of lutein and serum concentrations of zeaxanthin. Am. J. Clin. Nutr..

[66] Renzi L.M., Hammond B.R., Dengler M., Roberts R. (2012). The relation between serum lipids and lutein and zeaxanthin in the serum and retina: Results from cross-sectional, case control and case study designs. Lipids Health Dis..

[67] Conner W.E., Duell P.B., Kean R., Wang Y. (2007). The prime role of HDL to transport lutein into the retina: Evidence from HDL-defiocient WHAM chickens having a mutant ABCA1 transporter. Invest. Ophthalmol. Vis. Sci..

[68] Goodrow E.F., Wilson T.A., Houde S.C., Vishwanathan R., Scollin P.A., Handelman G., Nicholsi R.J. (2006). Consumption of one egg per day increases serum lutein and lipoprotein concentrations in older adults without altering serum lipid and lipoprotein cholesterol concentrations. J. Nutr..

[69] Blesso C.N., Andersen C.J., Bolling B.W., Fernandez M.L. (2013). Egg intake improves carotenoid status by increasing plasma HDL, cholesterol in adults with metabolic syndrome. Food Funct..

[70] Wang Y., Illingworth D.R., Conner S.L., Duell P.B., Conner W.E. (2010). Competitive inhibition of carotenoids transport and tissue concentrations by high supplements of lutein, zeaxanthin and beta-carotene. Eur. J. Nutr..

[71] Connolly E.E., Beatty S., Thurnham D.I., Loughman J., Howard A.N., Stack J., Nolan M. (2010). Augmentation of macula pigment following supplementation with all three macular carotenoids: An exploratory study. Curr. Eye Res..

[72] Wald G., Nathauson N., Jencks W.P., Tarr E. (1948). Crustacyanin, the blue carotenoid protein of the lobster shell. Biol. Bull..

[73] Bhosale P., Larson A.J., Frederick J.M., Southwick K., Thulin C.D., Bernstein P.S. (2004). Identification and characterization of a Pi isoform of glutathione s-transferase (GST1) as a zeaxanthin-binding protein in the macula of the human eye. J. Biol. Chem..

[74] Bhosale P., Li B., Sharifzadeh M., Gellermann W., Frederick J.M., Tscuchida K., Bernstein P.S. (2009). Purification and partial characterization of a lutein-binding protein from human retina. Biochemistry.

[75] Li B., Vachali P., Frederick J.M., Bersntein P.S. (2011). Identification of StARD3 as the lutein-binding protein in the macular of primate retina. Biochemistry.

[76] During A., Doraiswamy S., Harrison E.H. (2008). Xanthophylls are preferentially taken up compared with β-carotene by retinal cells via a SRBI-dependent mechanism. J. Lipid Res..

[77] Beatty S., Koh H., Phil M., Henson D.D., Boulton M. (2000). The role of oxidative stress in the pathogenesis of age-related macular degeneration. Surv. Ophthalmol..

[78] Chatterjee A., Milton R.C., Thyle S. (1982). Prevalence and etiology of cataracts in Punjab. Br. J. Ophthalmol..

[79] Das B.N., Thompson J.R., Patel R., Rosenthal A.R. (1990). The prevalence of age-related cataracts in the Asian community of Leicester: A community based study. Eye.

[80] Wolf-Schnurrbusch U.E.K., Roosli N., Weyermann E., Heldner M.R. (2007). Ethnic differences in macular pigment density and distribution. Invest. Ophthalmol..

[81] Sommer A., Tielsch J.M., Katz J., Quigly H.A., Gottsch J.D., Javitt J.C., Martone J.F., Royall R.M., Witt K.A., Ezrine S. (1991). Racial differences in the cause-specific prevalence of blindess in East Baltimore. N. Engl. J. Med..

[82] Dherani M., Murthy G.V., Gupta S.K., Young I.S., Maraini G., Camparini M., Priuce G.M., John N., Chakravarthy U., Fletcher A.E. (2008). Blood levels of vitamin C, carotenoids and retinol are inversely associated with cataract in a North Indian population. Invest. Ophthalmol. Vis. Sci..

[83] Moeller S.M., Voland R., Tinker L., Blodi B.A., Klein M.L., Gehrs M., Johnson E.J., Snodderly M., Wallace R.B., Chappell R.J. (2008). Associations between age-related nuclear cataract and lutein and zeaxanthin in the diet and serum in the carotenoids in the Age-Related Eye Disease Study, an ancillary study of the women’s Health Institute Initiative. Arch. Ophthalmol..

[84] Vu H.T., Robman L., Hodge A., McCarty C.A., Taylor H.R. (2006). Lutein and zeaxanthin and the risk of cataract: The Melbourne visual impairment project. Invest. Ophthalmol. Vis. Sci..

[85] Laitinen A., Laatikainen L., Harkanen T., Seppo K., Reunanen A., Aromaa A. (2010). Prevalence of major eye diseases and causes of visual impairement in the adult Finnish population, a nationwide population-based survey. Acta Ophtahlmol..

[86] Karppi J., Laukkanen J.A., Kurl S. (2012). Plasma lutein and zeaxanthin and the risk of age-related nuclear cataract among the elderly Finnish population. Br. J. Nutr..

[87] Augood C.A., Vingerling J.R., de Jong P.T., Chakravarthy U., Seland J., Soubrane G., Tomazzolis L., Topouzis F., Bentham G., Rahu M. (2006). Prevalence of age-related maculopathy in older Europeans: The European Eye Study (EUREYE). Arch. Ophthalmol..

[88] Li Y., Xu L., Jonas J.B., Yang H., Ma Y., Li J. (2006). Prevalence of age-related maculopathy in the adult population in China: The Beijing Eye Study. Am. J. Ophthalmol..

[89] Gupta S.K., Murthy G.V., Morrison N., Price G.M., Dherani M., John N., Fletcher A.E., Chakravarthy U. (2007). Prevalence of early and late age-related macular degeneration in a rural population in north India: The INDEYE feasibility study. Invest. Ophthalmol. Vis. Sci..

[90] Moon B.G., Joe S.G., Hwang J.-U., Kim H.K., Choe J., Yoon Y.H. (2012). Prevalence and risk factors of early-stage, age-related macular degeneration in patients examined at a health promotion centre in Korea. J. Korean Med. Sci..

[91] Mares-Perlman J.A., Klein R., Taylor A. (1999). Diet and Age-related Macular Degeneration. Nutritional and Environmental Influences on the Eye.

[92] Moeller S.M., Jacques P.F., Blumberg J.B. (2000). The potential role of dietary xanthophylls in cataract and age-related macular degeneration. J. Am. Coll. Nutr..

[93] Carpentier S., Knaus M., Suh M. (2009). Associations between lutein, zeaxanthin, and age-related macular degeneration. Crit. Rev. Food Sci. Nutr..

[94] Frégeau-Reid J., Abdel-Aal E.-S.M., Abdel-Aal E.-S.M., Wood P.J. (2005). Einkorn: A Potential Functional Wheat and Genetic Resource. Specialty Grains for Food and Feed.

[95] Abdel-Aal E.-S.M., Akhtar M.H. (2006). Recent advances in the analyses of carotenoids and their role in human health. Curr. Pharmaceut. Anal..

[96] Kean E.G., Hamaker B.R., Ferruzzi M.G. (2008). Carotenoids bioaccessibility from whole grain and degermed maize meal products. J. Agric. Food Chem..

[97] Abdel-Aal E.-S.M., Rabalski I. (2013). Effect of baking on free and bound phenolic acids in wholegrain bakery products. J. Ceral Sci..

[98] Abdel-Aal E.-S.M., Rabalski I. (2013). Antioxidant Properties of high-lutein grain-based functional foods in comparison with ferulic acid and lutein. Am. J. Biomed. Sci..

[99] De Oliveira G.P.R., Rodriguez-Amaya D.B. (2007). Processed and prepared corn products as sources of lutein and zeaxanthin: Compositional variation in the food chain. J. Food Sci..

